# Supplementary material for: Time to get our four priorities right: an 8-year prospective investigation of 1326 player-seasons to identify the frequency, nature, and burden of time-loss injuries in elite Gaelic football
Source: PeerJ. 2018 Jul 20;6:e4895. doi: 10.7717/peerj.4895 (PMC6055676; doi:10.7717/peerj.4895)
Supplement: Supplemental Information 2 — Incidence rate rate, IRR. Acute injury, A. Chronic/overuse injury, C/O. Non-contact injury, NC. Contact injury, C. [file peerj-06-4895-s002.doc]

**Supplementary Table 1 – Injury Incidence Across Age Groups**

|  | **Percentage of All Injuries** | **Incidence** | **Site IRR** | **Age IRR** | **Acute** | **Overuse/Chronic** | **A : C/O IRR** | **Contact** | **Non-Contact** | **NC : C IRR** |
| --- | --- | --- | --- | --- | --- | --- | --- | --- | --- | --- |
| **All Players** | **-** | **9.1 (8.6 - 9.5)** | **-** | **-** | **6.8 (6.4 - 7.2)** | **2.2 (2.0 - 2.4)** | **3.05 (2.72 - 3.42)** | **6.6 (6.3 - 7.0)** | **2.4 (2.1 - 2.6)** | **2.82 (2.52 - 3.15)** |
| Lower Limb | 79.9% (77.9 - 81.8) | 7.3 (6.9 - 7.7) | 4.00 (3.54 - 4.51) | - | 5.4 (5.0 - 5.7) | 1.9 (1.7 - 2.1) | 2.85 (2.52 - 3.22) | 5.9 (5.5 - 6.2) | 1.4 (1.2 - 1.5) | 4.33 (3.77 - 4.99) |
| Upper Limb | 10.5% (8.9 - 12.0) | 0.9 (0.8 - 1.0) | 0.11 (0.09 - 0.13) | - | 0.8 (0.7 - 1.0) | 0.1 (0.0 - 0.1) | 10.17 (4.40 - 23.52) | 0.3 (0.2 - 0.4) | 0.6 (0.5 - 0.7) | 0.44 (0.31 - 0.61) |
| Trunk | 6.6% (5.4 - 7.8) | 0.6 (0.5- 0.7) | 0.07 (0.06 - 0.09) | - | 0.3 (0.2 - 0.4) | 0.3 (0.2 - 0.4) | 0.92 (0.63 - 1.36) | 0.5 (0.4 - 0.6) | 0.1 (0.1 - 0.2) | 3.52 (2.22 - 5.60) |
| Head/Neck | 2.8% (2.0 - 3.6) | 0.3 (0.2 - 0.4) | 0.04 (0.03 - 0.05) | - | 0.3 (0.2 - 0.4) | - | - | 0.1 (0.0 - 0.1) | 0.3 (0.2 - 0.3) | 3.25 (1.30 - 4.80) |
| **18-20 Years** | **-** | **7.1 (6.0 - 8.3)** | **-** | **0.77 (0.65 - 0.91)** | **5.4 (4.3 - 6.4)** | **1.8 (1.2 - 2.4)** | **3.03 (2.07 - 4.44)** | **5.5 (4.4 - 6.5)** | **1.7 (1.1 - 2.2)** | **3.27 (2.22 - 4.83)** |
| Lower Limb | 79.8% (71.2 - 86.5) | 5.5 (4.4 - 6.5) | 3.27 (2.22- 4.83) | 0.73 (0.60 -0.89) | 3.9 (3.0 - 4.8) | 1.6 (1.0 - 2.1) | 2.48 (1.64 - 3.77) | 4.6 (3.7 - 5.6) | 0.9 (0.5 - 1.3) | 5.35 (3.19 - 8.98) |
| Upper Limb | 9.6% (4.8 - 15.4) | 0.7 (0.3 - 1.0) | 0.10 (0.06 - 0.18) | 0.70 (0.40 - 1.24) | 0.7 (0.3 - 1.0) | - | - | 0.2 (0.0 - 0.3) | 0.5 (0.2 - 0.8) | 0.30 (0.08 - 1.09) |
| Trunk | 5.8% (1.9 - 11.5) | 0.4 (0.1 - 0.7) | 0.06 (0.03 - 0.12) | 0.65 (0.32 - 1.34) | 0.2 (0.0 - 0.3) | 0.2 (0.0 - 0.4) | 0.75 (0.17 - 3.35) | 0.3 (0.1 - 0.5) | 0.1 (0.0 - 0.1) | 6.00 (0.72 - 49.83) |
| Head/Neck | 4.8% (1.0 - 9.6) | 0.7 (0.3 - 1.0) | 0.10 (0.06 - 0.18) | 2.48 (1.33 - 4.62) | 0.7 (0.3 - 1.0) | - | - | 0.4 (0.1 - 0.7) | 0.3 (0.0 - 0.5) | 1.60 (0.52 - 4.89) |
| **21-24 Years** | **-** | **7.8 (7.1 - 8.4)** | **-** | **0.78 (0.70 - 0.86)** | **5.9 (5.3 - 6.4)** | **1.9 (1.6 - 2.2)** | **3.11 (2.57 - 3.76)** | **5.6 (5.1 - 6.2)** | **2.1 (1.7 - 2.4)** | **1.09 (0.87 - 1.38)** |
| Lower Limb | 77.9% (73.7 - 81.7) | 6.1 (5.5 - 6.6) | 3.52 (2.89 - 4.29) | 0.75 (0.67 - 0.84) | 4.5 (4.0 - 5.0) | 1.6 (1.3 - 1.9) | 2.82 (2.28 - 3.48) | 5.0 (4.5 - 5.5) | 1.0 (0.8 - 1.3) | 3.59 (2.93 - 4.41) |
| Upper Limb | 11.6% (8.9 - 14.6) | 0.9 (0.7 - 1.1) | 0.13 (0.10 - 0.17) | 0.98 (0.72 - 1.34) | 0.8 (0.6 - 1.0) | 0.03 (0.0 - 0.1) | 10.17 (4.40 - 23.52) | 0.3 (0.2 - 0.4) | 0.6 (0.4 - 0.8) | 0.48 (0.28 - 0.80) |
| Trunk | 7.1% (4.8 - 9.4) | 0.6 (0.4 - 0.7) | 0.08 (0.06 - 0.11) | 0.89 (0.60 - 1.32) | 0.3 (0.2 - 0.4) | 0.3 (0.2 - 0.4) | 1.05 (0.57 - 1.94) | 0.2 (0.1 - 0.4) | 0.2 (0.1 - 0.3) | 2.08 (1.07 - 4.02) |
| Head/Neck | 3.2% (1.6 - 5.0) | 0.3 (0.1 - 0.4) | 0.03 (0.02 - 0.05) | 0.74 (0.43 - 1.30) | 0.3 (0.1 - 0.4) | - | - | - | 0.2 (0.1 - 0.4) | - |
| **25-29 Years** | **-** | **10.0 (9.3 - 10.8)** | **-** | **1.18 (1.07 - 1.30)** | **7.6 (6.9 - 8.3)** | **2.3 (2.0 - 2.7)** | **3.26 (2.72 - 3.89)** | **7.1 (6.5 - 7.8)** | **2.8 (2.4 - 3.2)** | **2.53 (2.13 - 2.99)** |
| Lower Limb | 81.5% (78.2 - 84.6) | 8.2 (7.5 - 8.9) | 4.53 (3.72 - 5.51) | 1.23 (1.10 - 1.37) | 6.2 (5.6 - 6.8) | 2.1 (1.7 - 2.4) | 3.03 (2.50 - 3.68) | 7.1 (6.4 - 7.7) | 2.0 (1.6 - 2.3) | 3.59 (2.93 - 4.41) |
| Upper Limb | 9.7% (7.2 - 12.3) | 1.0 (0.7 - 1.2) | 0.11 (0.08 - 0.14) | 1.13 (0.82 - 1.54) | 0.9 (0.7 - 1.1) | 2.0 (1.7 - 2.4) | 10.00 (4.32 - 23.14) | 0.4 (0.2 - 0.5) | 0.7 (0.5 - 0.9) | 0.48 (0.28 - 0.80) |
| Trunk | 5.7% (3.7 - 7.6) | 0.5 (0.4 - 0.7) | 0.06 (0.04 - 0.08) | 0.93 (0.63 - 1.38) | 0.3 (0.2 - 0.4) | 0.03 (0.0 - 0.1) | 1.00 (0.20 - 4.95) | 0.5 (0.3 - 0.7) | 0.1 (0.0 - 0.2) | 3.75 (1.72 - 8.18) |
| Head/Neck | 2.7% (1.4 - 4.3) | 0.3 (0.1 - 0.4) | 0.03 (0.02 - 0.04) | 0.81 (0.46 - 1.42) | 0.2 (0.1 - 0.4 | 0.3 (0.1 - 0.4) | - | - | 0.3 (0.2 - 0.4) | - |
| **>30 Years** | **-** | **13.1 (11.4 - 14.8)** | **-** | **1.51 (1.32 - 1.74)** | **9.2 (7.8 - 10.6)** | **3.8 (2.8 - 4.7)** | **4.38 (3.07 - 6.25)** | **10.5 (9.0 - 12.0)** | **2.6 (1.9 - 3.4)** | **4.00 (2.90 - 5.52)** |
| Lower Limb | 81.9% (76.3 - 87.0) | 10.7 (9.2 - 12.2) | 4.48 (3.21 - 6.25) | 1.55 (1.33 - 1.81) | 7.9 (6.5 - 9.2) | 3.0 (2.2 - 3.8) | 2.65 (1.93 - 3.65) | 9.0 (7.6 - 10.4) | 1.7 (1.0 - 2.2) | 5.48 (3.69 - 8.14) |
| Upper Limb | 7.3% (3.4 - 11.3) | 1.0 (0.5 - 1.4) | 0.08 (0.05 - 0.13) | 1.08 (0.65 - 1.78) | 0.9 (0.4 - 1.3) | 0.2 (0.0 - 0.4) | 5.00 (1.45 - 17.27) | 0.2 (0.0 - 0.5) | 0.7 (0.3 - 1.1) | 0.31 (0.10 - 0.94) |
| Trunk | 8.5% (4.5 - 13.0) | 1.1 (0.6 - 1.6) | 0.09 (0.06 - 0.14) | 2.01 (1.23 - 3.31) | 0.3 (0.0 - 0.5) | 0.6 (0.3 - 1.0) | 0.45 (0.16 - 1.31) | 1.0 (0.6 - 1.5) | 0.1 (0.0 - 0.2) | 7.00 (4.60 - 21.74) |
| Head/Neck | 2.3% (0.6 - 4.5) | 0.3 (0.0 - 0.5) | 0.02 (0.01 - 0.05) | 0.91 (0.36 - 2.28) | 0.2 (0.0 - 0.5) | - | - | 0.2 (0.0 - 0.4) | 0.2 (0.0 - 0.4) | 1.00 (0.20 - 4.95) |
