# Supplementary material for: Time to get our four priorities right: an 8-year prospective investigation of 1326 player-seasons to identify the frequency, nature, and burden of time-loss injuries in elite Gaelic football
Source: PeerJ. 2018 Jul 20;6:e4895. doi: 10.7717/peerj.4895 (PMC6055676; doi:10.7717/peerj.4895)
Supplement: Supplemental Information 3 — Presented with corresponding 95% confidence interval (CI). [file peerj-06-4895-s003.doc]

**Supplementary Table 2 – Time-Loss and Player Availability Across Age Groups**

|  | **Mean Time-Loss** | | | | |
| --- | --- | --- | --- | --- | --- |
|  | **All Players** | **18-20 Years** | **21-24 Years** | **25-29 Years** | **>30 Years** |
| **All Regions** | **25.7 (23.4 - 28.3)** | **22.4 (16.9 - 28.8)** | **23.8 (20.0 - 28.1)** | **28.5 (23.3 - 34.6)** | **28.7 (22.4 - 36.2)** |
| **Lower Limb** | 25.4 (22.8 - 28.5) | 24.0 (17.7 - 31.5) | 23.8 (19.5 - 28.7) | 28.1 (22.3 - 35.6) | 26.4 (19.8 - 34.7) |
| **Upper limb** | 28.8 (22.5 - 36.4) | 22.3 (10.3 - 35.0) | 26.4 (19.9 - 32.7) | 30.0 (17.3 - 48.5) | 41.6 (17.4 - 74.4) |
| **Trunk** | 28.9 (18.6 - 42.6) | 9.0 (4.4 - 14.2) | 25.0 (10.3 - 46.7) | 37.9 (14.1 - 71.7) | 40.3 (15.7 - 72.9) |
| **Head/Neck** | 13.3 (8.8 - 18.2) | 12.3 (5.0 - 20.0) | 8.1 (6.1 - 10.6) | 14.4 (6.3 - 23.8) | 8.5 (8.0 - 9.0) |
|  | **% of Unavailability** | | | | |
|  | **All Players** | **18-20 Years** | **21-24 Years** | **25-29 Years** | **>30 Years** |
| **Lower Limb** | 79.3% (77.1 - 80.8) | 82.7% (77.8 - 84.7) | 77.0% (75.3 - 80.4) | 80.8% (78.1 - 84.9) | 75.5% (71.0 - 79.5) |
| **Upper limb** | 11.1% (9.0 - 14.6) | 8.8% (2.8 - 14.4) | 12.7% (10.1 - 14.9) | 10.4% (5.8 - 15.0) | 11.0% (3.7 - 20.1) |
| **Trunk** | 7.8% (4.1 - 11.4) | 2.5% (0.4 - 4.2) | 7.2% (2.6 - 8.5) | 7.2% (2.4 - 13.8) | 11.6% (3.9 - 20.9) |
| **Head/Neck** | 1.5% (1.2 - 2.4) | 4.9% (1.3 - 8.2) | 1.2% (0.8 - 1.7) | 1.4% (0.5 - 2.3) | 6.1% (0.0 - 9.1) |
